# Comparative transcriptome analysis reveals key cadmium transport-related genes in roots of two pak choi (*Brassica rapa* L. ssp. *chinensis*) cultivars

**DOI:** 10.1186/s12864-017-3973-2

**Published:** 2017-08-08

**Authors:** Rugang Yu, Dan Li, Xueling Du, Shenglan Xia, Caifeng Liu, Gangrong Shi

**Affiliations:** College of Life Sciences, Huaibei Normal University, Huaibei, Anhui 235000 People’s Republic of China

**Keywords:** Pak choi, Root, Cd stress, Transcriptome, Transport

## Abstract

**Background:**

Cadmium translocation from roots to shoots is a complex biological process that is controlled by gene regulatory networks. Pak choi exhibits wide cultivar variations in Cd accumulation. However, the molecular mechanism involved in cadmium translocation and accumulation is still unclear. To isolate differentially expressed genes (DEGs) involved in transporter-mediated regulatory mechanisms of Cd translocation in two contrasting pak choi cultivars, Baiyewuyueman (B, high Cd accumulator) and Kuishan’aijiaoheiye (K, low Cd accumulator), eight cDNA libraries from the roots of two cultivars were constructed and sequenced by RNA-sequencing.

**Results:**

A total of 244,190 unigenes were obtained. Of them, 6827 DEGs, including BCd_10_ vs. BCd_0_ (690), KCd_10_ vs. KCd_0_ (2733), KCd_0_ vs. BCd_0_ (2919), and KCd_10_ vs. BCd_10_ (3455), were identified. Regulatory roles of these DEGs were annotated and clarified through GO and KEEG enrichment analysis. Interestingly, 135 DEGs encoding ion transport (i.e. ZIPs, P_1B_-type ATPase and MTPs) related proteins were identified. The expression patterns of ten critical genes were validated using RT-qPCR analysis. Furthermore, a putative model of cadmium translocation regulatory network in pak choi was proposed.

**Conclusions:**

High Cd cultivar (Baiyewuyueman) showed higher expression levels in plasma membrane-localized transport genes (i.e., *ZIP2*, *ZIP3*, *IRT1*, *HMA2* and *HMA4*) and tonoplast-localized transport genes (i.e., *CAX4*, *HMA3*, *MRP7*, *MTP3* and *COPT5*) than low Cd cultivar (Kuishan’aijiaoheiye). These genes, therefore, might be involved in root-to-shoot Cd translocation in pak choi.

**Electronic supplementary material:**

The online version of this article (doi:10.1186/s12864-017-3973-2) contains supplementary material, which is available to authorized users.

## Background

Soil contamination with cadmium (Cd) has long been a major ecological concern worldwide because of the potential threat to all organisms. Cd is readily transferred from contaminated soil to plants and accumulates in different organs [1–3]. The accumulation of Cd in plants may lead to severe toxicity in both plants and animals/humans [4]. To prevent this negative effect, two strategies have been proposed: (i) reducing Cd concentration in contaminated soils using phytoremediation and chemical remediation [4, 5]; and (ii) reducing Cd concentration in edible parts of plants by breeding low-Cd accumulation cultivars. For these purposes, understanding the molecular mechanisms underlying Cd uptake, translocation and accumulation in plants is critically important.

Several processes are involved in the distribution and translocation of Cd in plant roots [6–8], including (i) transporter-mediated Cd translocation via symplasmic or apoplastic pathway; (ii) deposition of Cd in the cell wall; and (iii) sequestration of Cd-chelates in the vacuole. Most of these processes are mediated by transport-related genes. In rice, Satoh-Nagasawa et al. [9] report that loss of *OsHMA2* function in insertion mutant results in decreased leaf and grain Cd concentrations. *OsHMA3* plays a role in the sequestration of Cd into vacuoles in root cells [10, 11], and *OsHMA3* expression in the Cd-sensitive Δycf1 mutant can cause a wild-type tolerance [10]. Furthermore, the member of Nramp family in rice, *OsNramp1* [12] and *OsNramp5* [13–15] were reported to be Cd transporter involved in the root uptake of Cd from the medium. These findings suggested that a number of metal-regulated transporter proteins could participate in cellular Cd uptake and translocation within plants.

High-throughput cDNA sequencing (RNA-seq) is a recently developed approach to transcriptome profiling that has been used to discover and characterize genes, analyze functional gene variation, and identify and quantify rare transcripts [16, 17]. Using this approach, many Cd stress-related genes have been identified in *Arabidopsis* [18], ramie [19], radish [17] and *Viola yedoensis* [20]. In addition, comparative transcriptomic analyses have been used for revealing the mechanisms of Cd accumulation in plants [21–23]. These studies have greatly improved our understanding of the molecular regulation mechanisms underlying Cd tolerance and accumulation.

Pak choi (*Brassica rapa* L. ssp. *chinensis*) is an important leafy vegetable crop. Variability among pak choi cultivars in Cd concentration has been reported [24, 25]. However, the molecular mechanism of Cd accumulation in pak choi is not well understood [23]. Although a fraction of DEGs related to Cd stress has been identified in pak choi [23], it might not broadly and deeply reveal the mechanism of Cd uptake and translocation.

Here, a comparative transcriptome analysis was performed using RNA-seq technology. The aims were: (i) to obtain assembled unigenes from pak choi root transcriptome; (ii) to investigate the gene expression patterns in the roots of two distinct pak choi genotypes under long-term Cd exposure; and (iii) to identify DEGs involved in Cd-transport regulatory networks.

## Results

### Illumina sequencing and transcriptome assembly

**Fig. 1:**
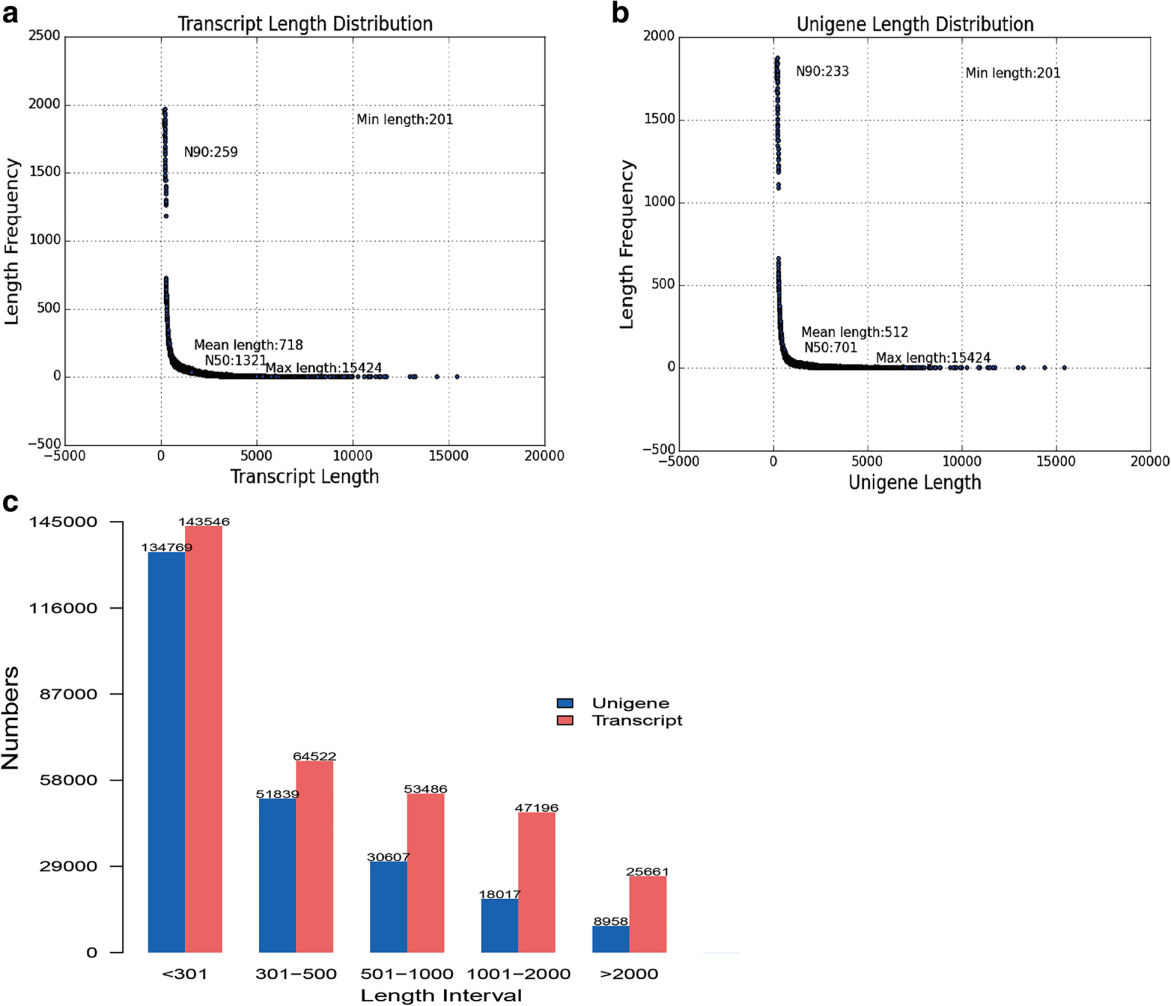
The length distribution of assembled transcripts (**a** and **c**) and unigenes (**b** and **c**) from pak choi transcriptome. Transcripts and unigenes were assembled from raw sequence data after reads with adapters, reads with ploy-N and low quality reads were removed

A total of 119.6 × 10^6^, 114.5 × 10^6^, 105.8 × 10^6^ and 118.3 × 10^6^ raw reads were generated from two replicate libraries for BCd_0_, BCd_10_, KCd_0_ and KCd_10_, respectively (Table 1). After filtering the reads with low quality and adapters, the percentages of clean reads in all eight transcriptomes were all above 95.99%, and the proportion of Q20 bases for the clean reads was above 95.93% for each library (Table 1). Using the Trinity program, a total of 334,411 putative transcripts were obtained, with an average length of 718 bp and an N50 of 1321 bp, and transcripts with lengths of more than 500 bp accounted for about 37.78% of all transcripts (Fig. 1a and c). After comparing the different transcripts representing one unigene, the longest transcript for each locus was selected as the unigene. In all, 244,190 unigenes were obtained as reference transcripts of pak choi. The mean length was 512 bp, and unigenes with lengths of more than 500 bp accounted for about 23.58% of all unigenes (Fig. 1b and c).

**Table 1:** Overview of raw and clean reads in control (Cd_0_) and Cd (Cd_10_) treatments for two pak choi cultivars

Libraries	Raw Reads	Clean Reads	Clean reads in raw reads (%)	Clean Bases	Error rate (%)	Q20 (%)	GC Content (%)
B1_Cd_0_	66,471,522	64,624,428	97.22	9.69G	0.02	96.65	46.61
B2_Cd_0_	53,091,622	51,435,618	96.88	7.72G	0.02	96.31	46.58
B1_Cd_10_	64,130,424	61,972,662	96.64	9.30G	0.02	96.44	46.66
B2_Cd_10_	50,319,622	48,490,708	96.37	7.27G	0.02	95.93	46.70
K1_Cd_0_	55,192,566	53,300,252	96.57	8.00G	0.02	96.18	47.06
K2_Cd_0_	50,640,386	49,035,860	96.83	7.36G	0.02	96.61	46.86
K1_Cd_10_	55,041,300	52,835,866	95.99	7.93G	0.02	95.98	46.58
K2_Cd_10_	63,262,852	61,379,256	97.02	9.21G	0.02	96.60	46.65

### Functional annotation and classification of non-redundant unigenes

To investigate potential functions of the assembled unigenes, sequence similarity searches of 244,190 unigenes were performed in the public databases. In total, 142,631 unigenes were annotated representing 41.59% of the assembled unigenes. In the Nr, Pfam, GO, KOG and KEGG databases, 108,001, 99,209, 100,852, 61,894 and 53,311 unigenes were aligned, respectively (Table 2).

**Table 2:** Summary of the functional annotation of assembled unigenes in pak choi

Public database	Number of unigenes	Percentage (%)
Annotated in Nr	108,001	44.22
Annotated in Nt	2381	0.97
Annotated in KEGG	53,311	21.83
Annotated in Swiss Prot	93,001	38.08
Annotated in Pfam	99,209	40.62
Annotated in GO	100,852	41.30
Annotated in KOG	61,894	25.34
Annotated in all Databases	490	0.20
Annotated in at least one Database	142,631	58.40
Total Unigenes	244,190	100

**Fig. 2:**
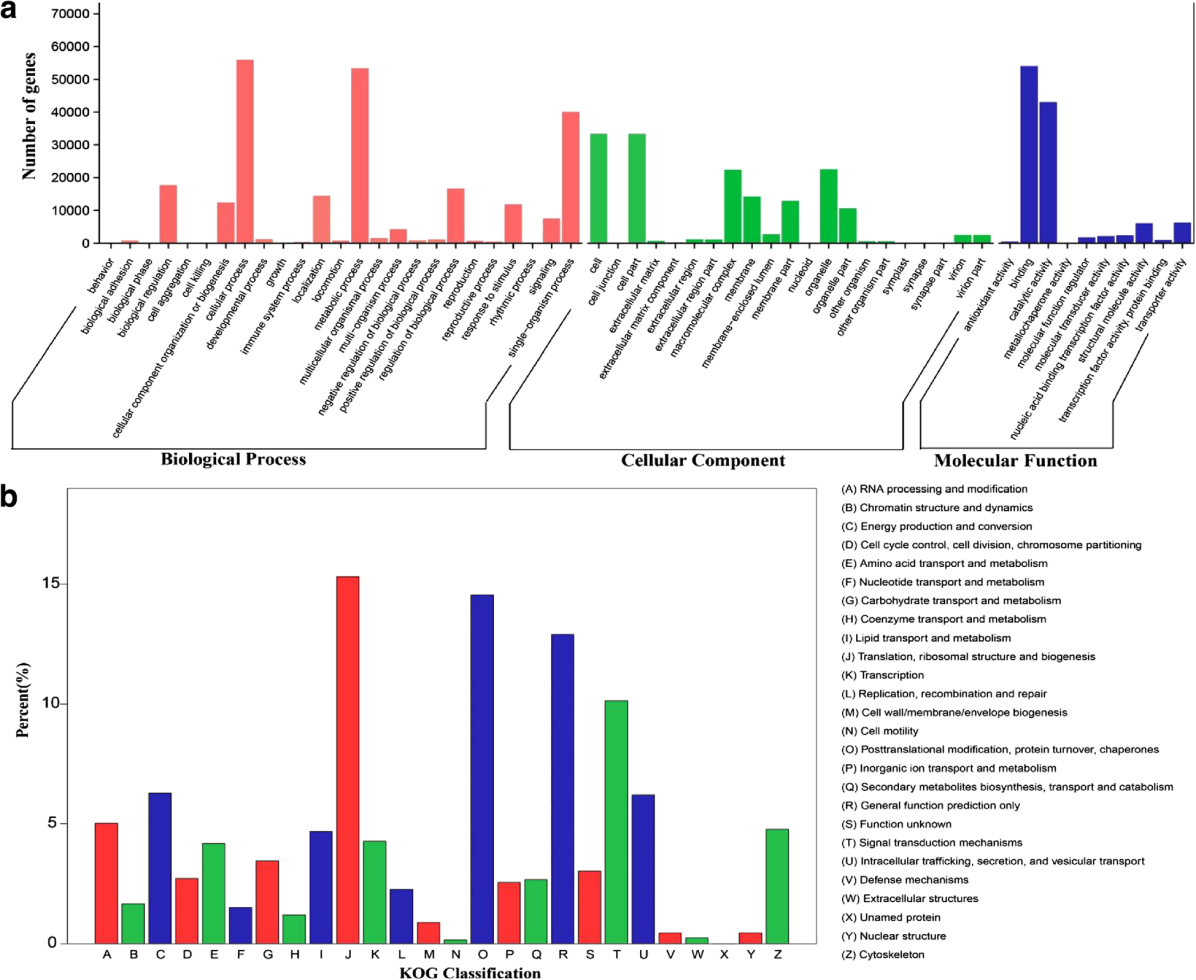
Gene Ontology (GO) (**a**) and histogram presentation of euKaryotic Ortholog Groups (KOG) (**b**) classifications for assembled unigenes of pak choi transcriptome. **a** The x-axis represents the GO term; the y-axis denotes the number of unigenes; **b** The capital letters in x-axis show the KOG categories as listed on the right, and the y-axis indicates the number of unigenes in each category

GO assignments were used to classify the functions of all predicted unigenes based on the annotations from the Nr and Pfam databases. In total, 100,852 unigene sequences (41.3%) were categorized into 56 functional groups consisting of 25 biological process, 21 cellular components and 10 molecular function subcategories (Fig. 2a).

The sequence similarity search was performed against the KOG databases to obtain the functional annotations of assembled unigenes. 61,894 unigene sequences (25.34%) with significant homology were assigned to 26 KOG categories (Fig. 2b). Among them, the five largest groups included ‘translation, ribosomal structure and biogenesis’ (9479, 15.31%), ‘posttranslational modification, protein turnover, chaperones’ (9004, 14.55%), ‘general function prediction’ (7987, 12.90%), ‘signal transduction mechanisms’ (6279, 10.14%) and ‘energy production and conversion’ (3890, 6.28%).

**Fig. 3:**
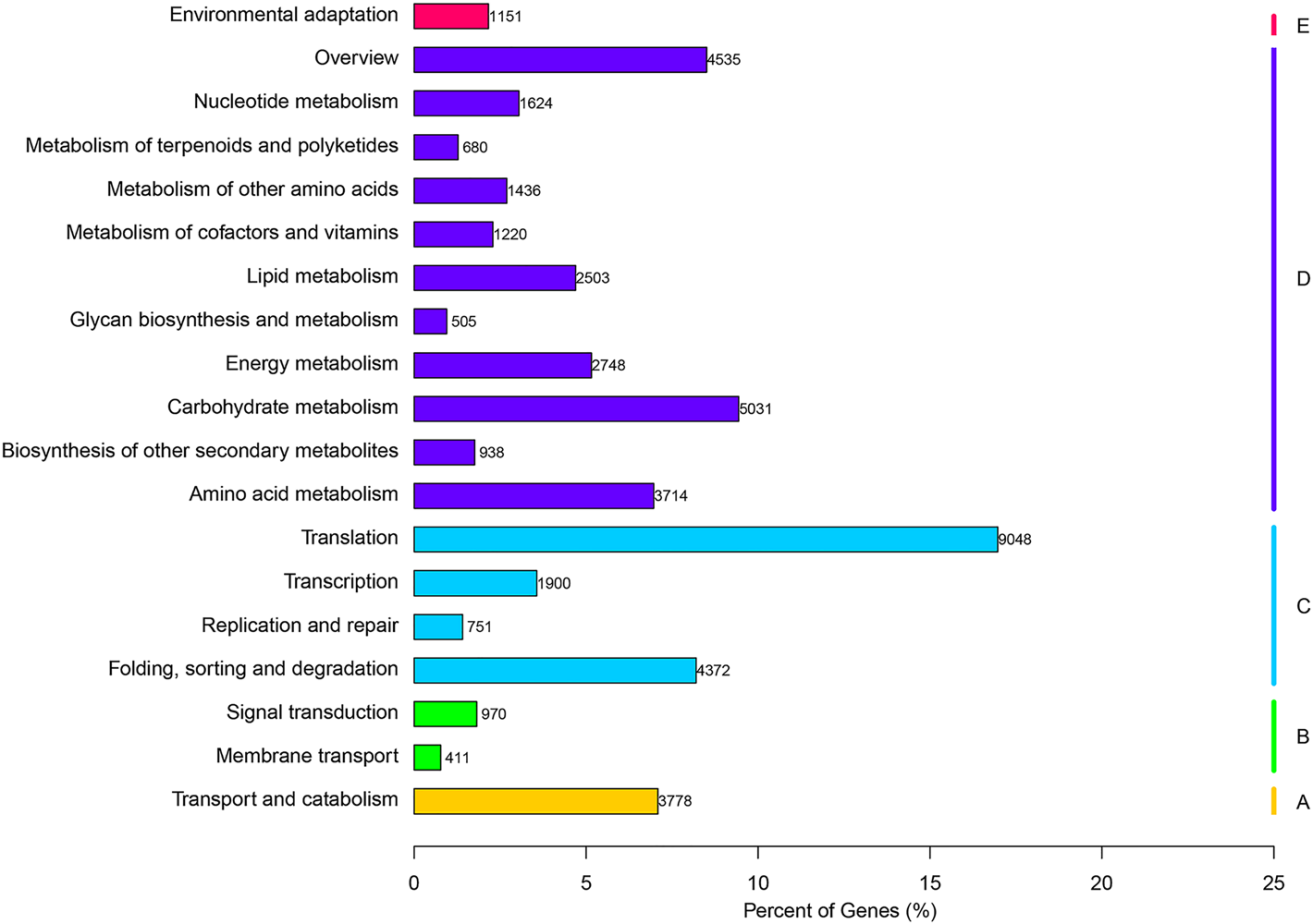
Functional classification and pathway assignment of assembled unigenes by KEEG. The unigenes were assigned pathways belonging to five main categories: Cellular processes (**a**); Environmental information processing (**b**); Genetic information processing (**c**); Metabolism (**d**); Organismal Systems (**e**), including 19 sub-categories

To further characterize molecular-level gene functions in terms of biological system networks, the assembled unigenes were mapped against the KEGG protein database. 53,311 unigenes with significant matches were assigned to 132 KEGG pathways, of which the top five were ‘ribosome’, ‘carbon metabolism’, ‘biosynthesis of amino acids’, ‘protein processing in endoplasmic reticulum’ and ‘RNA transport’ (Additional file 1: Table S1). These pathways belonged to five main categories that could be further divided into 19 subcategories (Fig. 3). Among them, translation was the dominant pathway (9048 unigenes, 16.97%), followed by carbohydrate metabolism (5031, 9.44%), overview (4535, 8.51%), folding, sorting and degradation (4372, 8.20%) and transport and catabolism (3778, 7.09%).

### Identification and functional annotation of DEGs

**Fig. 4:**
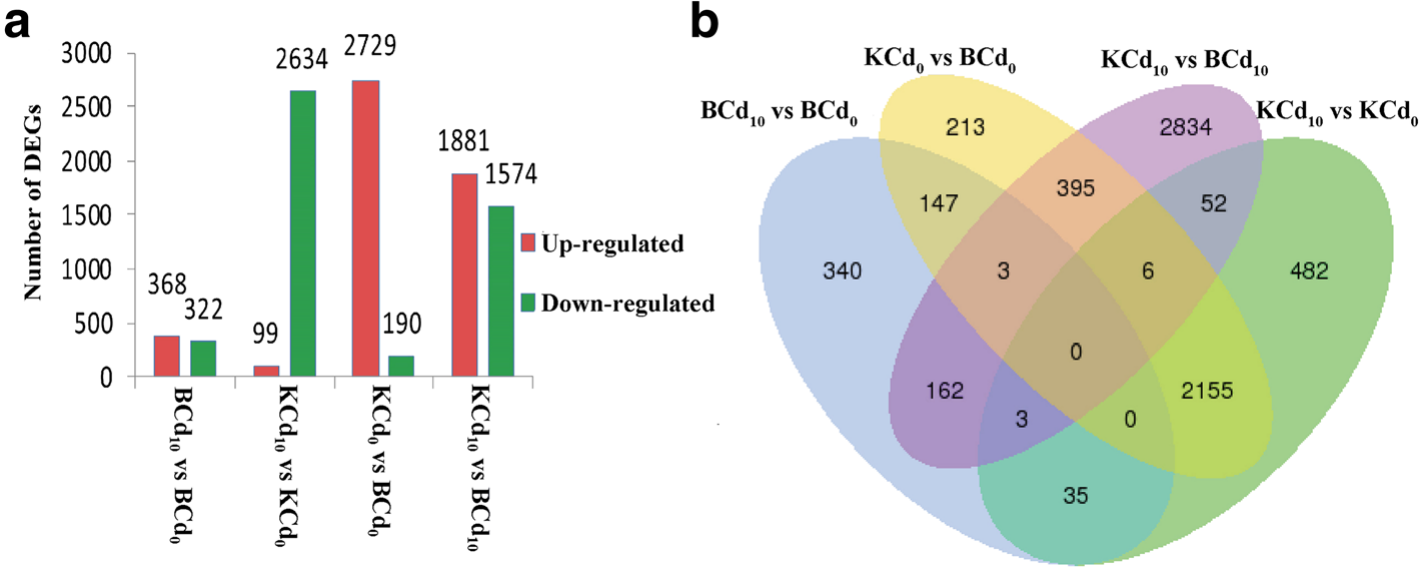
Analysis of DEGs following Cd exposure in two pak choi cultivars. **a** Numbers of DEGs in each comparison. Up- and down-regulated genes are shown in *red* and *green*, respectively. **b** Venn diagrams of DEGs among four comparisons. This shows the numbers of unigenes from multiple comparisons among the four groups: BCd_0_, BCd_10_, KCd_0_ and KCd_10_

A total of 6827 unigenes were found to have significant differential expression in the four comparisons, including BCd_10_ vs. BCd_0_ (690 unigenes), KCd_10_ vs. KCd_0_ (2733 unigenes), KCd_0_ vs. BCd_0_ (2919 unigenes) and KCd_10_ vs. BCd_10_ (3455 unigenes) (Additional file 2: Table S2; Fig. 4a). The Venn diagram indicates the distribution of DEGs among the four comparisons (Fig. 4b). Among of these DEGs, up-regulated DEGs accounted for 53.33% of BCd_10_ vs. BCd_0,_ 93.49% of KCd_0_ vs. BCd_0_, and 54.44% of KCd_10_ vs. BCd_10_ DEGs, whereas only 3.62% of DEGs were up-regulated in KCd_10_ vs. KCd_0_ (Fig. 4a **)**. There were 2239 DEGs that showed similar expression patterns in the two cultivars, while 2076 DEGs showed opposite expression patterns (Additional file 2: Table S2). Furthermore, 2265 DEGs were specifically regulated by Cd in Kuishan’aijiaoheiye, while only 137 specific regulated DEGs were found in Baiyewuyueman (Additional file 2: Table S2).

GO and pathway enrichment analysis were conducted to gain functional annotations of the DEGs. A total of 70, 67, 93 and 46 terms were significantly enriched for DEGs in BCd_10_ vs. BCd_0_, KCd_10_ vs. KCd_0,_ KCd_0_ vs. BCd_0_, and KCd_10_ vs. BCd_10_, respectively, and of these, 62.86%, 2.99%, 83.87% and 76.09%, respectively, were up-regulated (Additional file 3: Table S3). In Baiyewuyueman, the predominant enriched GO terms of the Cd-induced up-regulated DEGs were related to cellular process (GO: 0009987), metabolic process (GO: 0008152) and organic substance metabolic process (GO: 0071704) in biological processes. Among the down-regulated DEGs, the mainly enriched GO terms were related to transcription, DNA-templated (GO: 0006351), nucleic acid-templated transcription (GO: 0097659) and RNA biosynthetic process (GO: 0032774) in biological processes. In Kuishan’aijiaoheiye, the majority of annotated DEGs (97.01%) were down-regulated by Cd. The predominant enriched GO terms of these DEGs were involved in biological processes including cellular process (GO: 0009987), single-organism cellular process (GO: 0044763) and cell (GO: 0005623). Compared with Baiyewuyueman, some predominantly enriched GO terms of down-regulated DEGs related to biological processes such as response to oxidative stress (GO: 0006979), cell wall organization (GO: 0071555), glucan metabolic process (GO: 0044042) and obsolete peroxidase reaction (GO: 0006804), were observed in Kuishan’aijiaoheiye under Cd treatment (Additional file 3: Table S3).

Additionally, a total of 44 and 40 significantly enriched pathways were obtained for up and down-regulated DEGs, respectively (Additional file 4: Table S4). As expected, the DEGs annotated in the pathway showed different effects in Baiyewuyueman and Kuishan’aijiaoheiye exposed to Cd. The most significantly enriched pathway annotations for Baiyewuyueman were up-regulated DEGs under Cd stress, such as ‘ribosome’ (74 unigenes), ‘legionellosis’ (10 unigenes), ‘antigen processing and presentation’ (7 unigenes) and ‘carbon fixation in photosynthetic organisms’ (6 unigenes) etc. (Additional file 4: Table S4). However, the most significantly enriched annotations for Kuishan’aijiaoheiye were down-regulated DEGs under Cd stress. The predominantly enriched pathways for the down-regulated DEGs were involved in ‘purine metabolism’ (41 unigenes), ‘spliceosome’ (29 unigenes), ‘rap1 signaling pathway’ (29 unigenes), ‘oxytocin signaling pathway’ (29 unigenes) and ‘leukocyte transendothelial migration’ (29 unigenes) (Additional file 4: Table S4). Furthermore, some Cd stress-related pathways, such as glutathione metabolism (ko00480), ascorbate and aldarate metabolism (ko00053), glycine, serine and threonine metabolism (ko00260) and plant hormone signal transduction (ko04075) are significantly enriched for the up-regulated DEGs in KCd_10_ vs. BCd_10_.

### DEGs involved in Cd transport

**Fig. 5:**
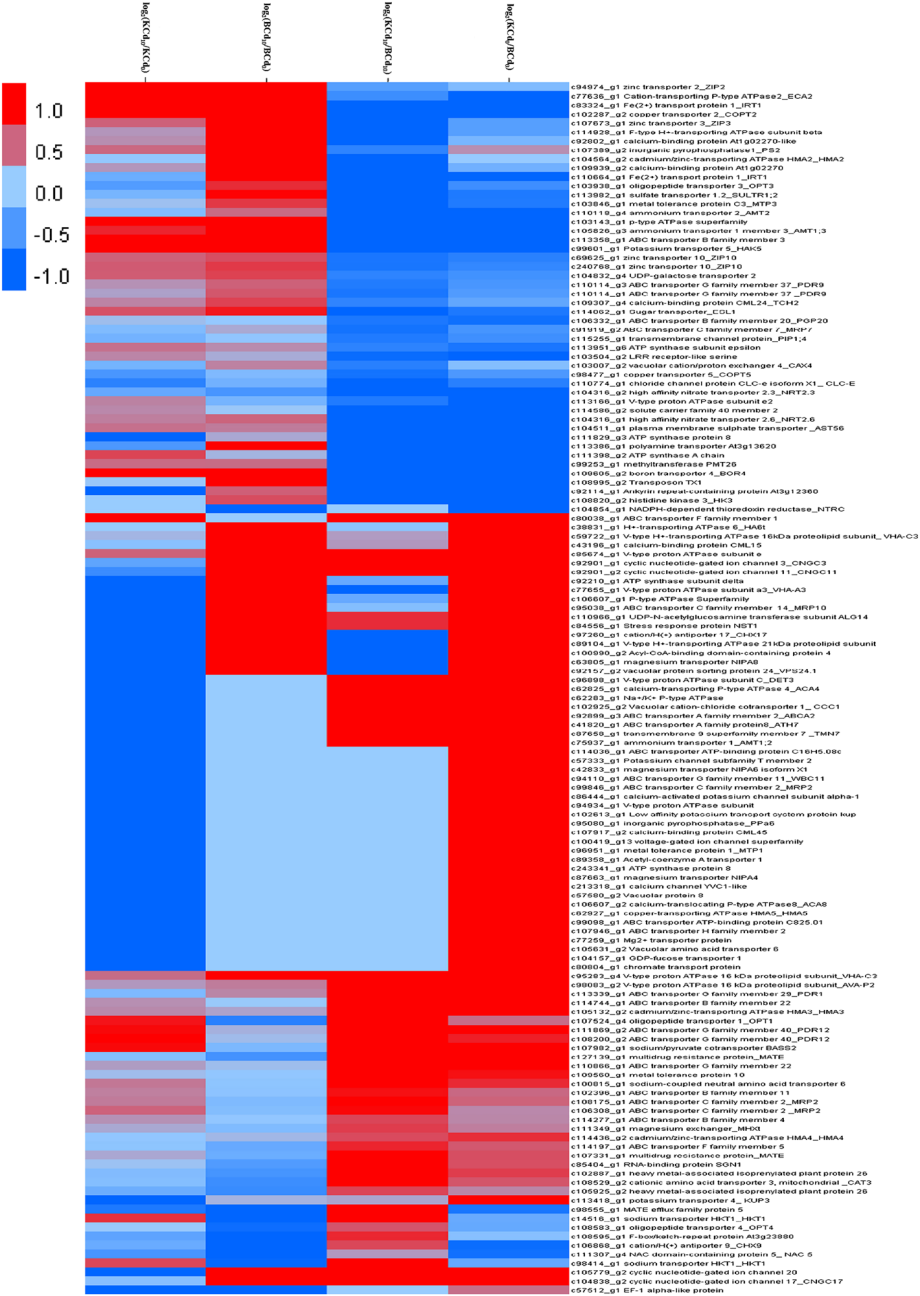
Hierarchical cluster analysis of transport-related genes expression profiles among four comparisons. The heat map represents cluster analysis of 135 differentially expressed unigenes. The log_2_ of readcount ratio for each unigene was used for hierarchical analysis in each of four comparisons: BCd_10_ vs. BCd_0_, KCd_0_ vs. KCd_10_, KCd_0_ vs. BCd_0_, and KCd_10_ vs. BCd_10_. *Red* and *blue* colors indicate up- and down-regulated unigenes in each comparison, respectively. The *bar* represents the scale of relative gene expression

In the four comparisons, 135 DEGs were identified as having high similarity with diverse transporters such as zinc transporters (e.g. ZIP2, ZIP3 and ZIP10), Fe^2+^ transport protein 1 (IRT1), P_1B_-ATPase (e.g. HMA2, HMA3, HMA4 and HMA5), ABC transporters, vacuolar cation/proton exchanger 4 (CAX4), oligopeptide transporter (OPT) families and metal tolerance proteins (MTPs) (Fig. 5; Additional file 5: Table S5). Of 135 DEGs, 11 and 4 unigenes were up-regulated by Cd, while 5 and 44 unigenes were down-regulated in Baiyewuyueman and Kuishan’aijiaoheiye respectively. All unigenes belonging to ZIPs, high affinity nitrate transporter 2.6 (*NRT2.6*) and *HMA3* were tended to be up-regulated in both cultivars compared to their respective controls. All unigenes encoding *CAX4* and cyclic nucleotide gated channels (CNGCs) were up-regulated in Baiyewuyueman and down-regulated in Kuishan’aijiaoheiye compared to their respective controls. Based on pairwise comparisons, members within the some families such as OPTs, ATP synthases, V-type proton ATPase (V-ATPase), P-type ATPase superfamily (P-ATPase), inorganic pyrophosphatase (PPase), cadmium/zinc/copper-transporting ATPase (HMAs) and metal tolerance proteins (MTPs), had considerably variable in expression pattern in the four groups. Moreover, the same member within the same pairwise comparison also had a different expression pattern; for example, two unigenes (c127139_g1 and c107331_g1) encoding a multidrug resistance protein (MRP) were not consistent with the pattern of expression in KCd10 vs. KCd0. In addition, for KCd10 vs. BCd10, all unigenes encoding ZIPs, IRT1, HA6t, NRTs, P-type ATPase superfamily (P-ATPase) and copper transporters (COPTs) were down-regulated. The majority of unigenes belonging to ABC transporters (68.4%) and calcium-binding proteins (CMLs, 75.0%) were identified as down-regulated between Baiyewuyueman and Kuishan’aijiaoheiye in Cd treatments. Surprisingly, several critical Cd-related transporters such as natural resistance-associated macrophage proteins (Nramps), zinc induced facilitators (ZIFs) and ABC transporter G family member 35 (PDR8) were similar in the four groups.

### RT-qPCR validation

**Fig. 6:**
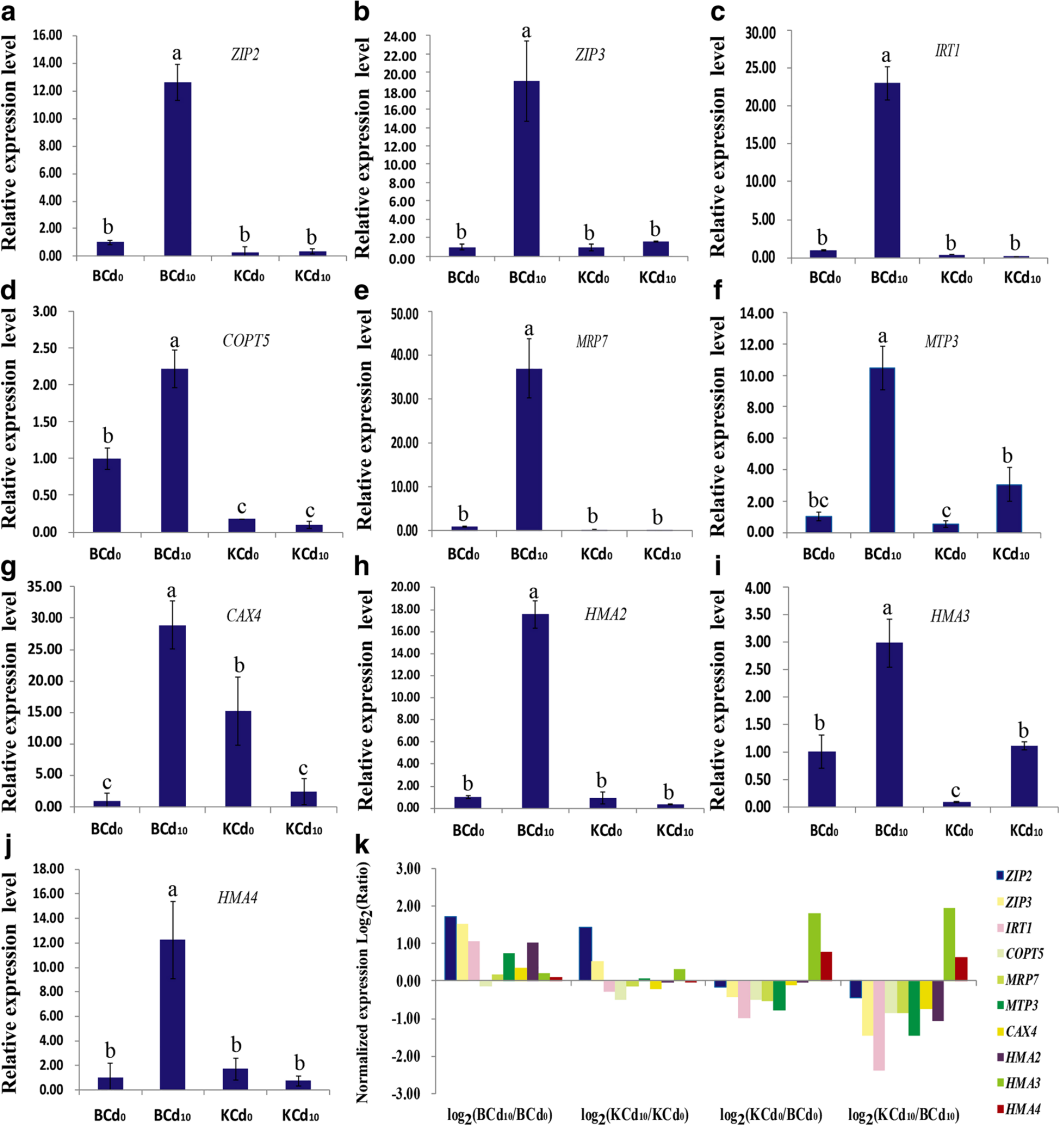
RT-qPCR validation of selected ten Cd transporter-related DEGs under the control and Cd-treated in roots of two pak choi cultivars. The relative expression levels of each selected gene was determined by 2^-ΔΔ*C*T^. Each bar represents the mean ± STD of triplicate assays. Values with different letters indicate significant differences at *P* < 0.05 according to Duncan’s multiple range tests. The ten DEGs including zinc-regulated transporter 2/3 (ZIP2/3), iron-regulated transporter 1 (IRT1), copper transporter 5 (COPT5), multidrug resistance protein 7 (MRP7), metal tolerance protein 3 (MTP3), cation exchanger 4 (CAX4) and cadmium/zinc/copper-transporting ATPase HMA 2/3/4 (HMA2/3/4), were analyzed by RT-qPCR (**a-j**) and comparized with expression profile by RNA-seq (**k**)

Ten transport genes including *ZIP2*, *ZIP3*, *IRT1*, *MRP7*, *MTP3*, *COPT5*, *CAX4*, *HMA2*, *HMA3* and *HMA4* were identified to relevant to the regulation of Cd translocation. The expression of these genes and their biological functions are listed in Table 3. These genes were also used for confirming the reliability of RNA sequencing results by RT-qPCR. As shown in Fig. 6, the relative expression levels of ten genes were significantly higher in BCd_10_ than in KCd_10_, and were dramatically up-regulated in BCd_10_ vs. BCd_0_. Two strongly up-regulated genes (*MTP3* and *HMA3*) and one significantly down-regulated gene (*CAX4*) were observed in KCd_10_ vs. KCd_0_. Meanwhile, the relative expression of *ZIP2*, *ZIP3*, *IRT1*, *COPT5*, *MRP7*, *HMA2* and *HMA4* were similar between the control and Cd-treatment in Kuishan’aijiaoheiye. The expression patterns from RT-qPCR were similar to those of the RNA-Seq-based gene expression patterns (Fig. 6k). However, three genes including *COPT5* (BCd_10_ vs. BCd_0_), *HMA3* and *HMA4* (KCd_10_ vs. BCd_10_) did not show consistent expression levels between RT-qPCR and Illumina sequencing data (Fig. 6). The discrepancies may be result from different sensitivity of the two techniques.

**Table 3:** Critical DEGs involved in Cd transport in roots of two pak choi genotypes from this study and their putative roles from the literature

Unigene ID	NR Description	Gene name	log_2_Ratio^a^	*p*-adjusted	log_2_Ratio^b^	*p*-adjusted	log_2_Ratio^c^	*p*-adjusted	Reference
Plasma membrane-localized transporters									
c107673_g1	zinc transporter 3	*ZIP3*	1.5388	0.057222	0.5221	1	−1.4552	0.023467	[28, 41]
c94974_g1	zinc transporter 2	*ZIP2*	1.7162	9.0557E-09	1.4462	0.00008204	−0.4343	0.59414	[28, 41]
c83324_g1	Fe^2+^ transport protein 1	*IRT1*	2.5396	0.12262	1.4140	1	−2.8878	0.013235	[42]
c110664_g1	Fe^2+^ transport protein 1	*IRT1*	1.0774	0.44309	−0.2944	1	−2.3726	0.00015383	[42]
c104564_g2	cadmium/zinc-transporting ATPase HMA2	*HMA2*	1.0251	0.30443	−0.0342	1	−1.0753	0.046561	[9, 38]
c114436_g2	cadmium/zinc-transporting ATPase HMA4	*HMA4*	0.0924	1	−0.0435	1	0.6318	0.026928	[34]
Tonoplast-localized transporters									
c91919_g2	ABC transporter C family member 7	*MRP7*	0.1662	1	−0.1586	1	−0.8707	0.033998	[31]
c98477_g1	copper transporter 5	*COPT5*	−0.1600	1	−0.5146	0.81771	−0.8643	0.0018347	[55]
c103007_g2	vacuolar cation/proton exchanger 4	*CAX4*	0.3642	1	−0.2353	1	−0.7353	0.02893	[40]
c105132_g2	cadmium/zinc-transporting ATPase HMA3	*HMA3*	0.2008	1	0.3235	1	1.9350	7.0614E-09	[10]
c103846_g1	metal tolerance protein C3	*MTP3*	0.7265	0.79549	0.0521	1	−1.4629	0.002138	[53]

^a^log_2_(BCd_10__readcount/BCd_0__readcount)

^b^log_2_(KCd_10__readcount/KCd_0__readcount)

^c^log_2_(KCd_10__readcount/BCd_10__readcount)

## Discussion

### Comparative transcriptome analysis of two pak choi cultivars

RNA-Seq approach has been successfully and increasingly used for revealing the mechanisms of Cd resistance and accumulation in plants [21, 12, 26]. According to a recent report, 59,271 unigenes were identified in pak choi, of which 44,539 were functionally annotated and from which many DEGs were obtained [23]. The result provided valuable information for understanding the molecular mechanism of Cd resistance. However, owing to the limitations of genotype and development stages selected, the results could not reflect the overall expression profiles of Cd stress-related genes. In this study, a total of 244,190 unigenes were obtained from eight samples by RNA sequencing and assembly (Table 2), with 142,631 (41.59%) of them successfully annotated by BLAST comparisons with the public databases, which is a greater number than previous reported for pak choi [23]. The results provide a foundation for further investigation of Cd stress mechanisms and identification of novel genes in pak choi. In addition, the large numbers of un-annotated unigenes could be the result of several causes, including novel genes specifically expressed in pak choi roots, the lack of information on the genomes or transcriptomes of pak choi, and other technical or biological biases, such as assembly parameters.

Previous study indicated that the shoot Cd concentration was higher in Baiyewuyueman than in Kuishan’aijiaoheiye under the same Cd condition [25]. In this study, a total of 6827 unigenes were identified as DEGs in four comparisons (Additional file 2: Table S2). Among these DEGs, 368 (368/690, 53.33%) were significantly up-regulated in BCd_10_ vs. BCd_0,_ and only 99 (99/2733, 3.62%) in KCd_10_ vs. KCd_0_. These results suggest that Baiyewuyueman could more effectively activate gene expression under Cd treatment. In addition, 2265 DEGs were specifically regulated by Cd in Kuishan’aijiaoheiye, while only 137 DEGs in Baiyewuyueman (Additional file 2: Table S2). Furthermore, we found that 2239 overlapping DEGs showed similar expression patterns in Kuishan’aijiaoheiye and Baiyewuyueman under Cd stress (Additional file 2: Table S2). These DEGs are not the key genes for regulating Cd tolerance and accumulation in the two cultivars. Besides, 2076 unigenes showed opposing expression patterns in Cd treatment (Additional file 2: Table S2). These results indicate that the two cultivars differed in the molecular mechanisms of Cd response. Additionally, many unigenes were annotated in categories and pathways related to cellular and metabolic processes (Additional file 3: Table S3; Additional file 4: Table S4). These data could be useful for genomic studies and the identification of functional genes in pak choi.

### Key genes related to Cd transport in root cells

Cd is mainly transported across the plasma membrane and tonoplast by diverse influx and efflux families of transporters and cation channels in root cells [27, 28]. To date, numerous transporter genes such as ZIP-IRT1 transporters [28, 29], ABC transporters [30, 31], CAXs [32], OPTs [7], COPTs [33] and P_1B_-ATPase (e.g. HMA2, HMA3 and HMA4) [10, 11, 34], are already known to be involved in Cd transport in plants. Moreover, the role of cation channels (CNGCs and calcium channels) in Cd^2+^ transport to root cells have been reported [7, 28]. Studies have also demonstrated that multiple transporters play an important role in Cd responses in plants [17, 21, 23]. Xu et al. [17] identified 24 HM-mediated peptides and transporters as Cd-stressed responsive genes in radish, including members of the CAX2, P-type ATPase and ZIP families. Zhou et al. [23] identified 63 DEGs belonging to eight GO terms involved in cation transport, which contributed to the genotype differences in Cd accumulating abilities in pak choi. In the current study, many metal transporters and cation channels belonging to various families were also found to be differentially expressed in all pairwise comparisons (Additional file 5: Table S5). Interestingly, 44 unigenes encoding heavy metal transporters, such as *ZIP3*, *ZIP10*, *IRT1*, *OPT3*, *MRP7*, *PDR9*, *CAX4*, *HMA2* and *COPT5*, showed higher expression in Cd-treated Baiyewuyueman than in Kuishan’aijiaoheiye. However, other heavy metal transporters including *HMA* (3, 4), *PDR* (1, 12), *MRP2*, *HKT1* and *OPT1*, displayed lower expression levels in Baiyewuyueman roots than in Kuishan’aijiaoheiye (Additional file 5: Table S5). In addition, Zhou et al. [23] found that *PDR8* was highly expressed in LAJK compared to HAJS, which may contribute to the low Cd uptake and accumulation in LAJK. *OsNRAMP1* and *OsNRAMP5* were reported to be Cd transporter involved in the root uptake of Cd from the medium [12–15]. However, no cultivar difference was detected in the expression of *PDR8 and NRAMPs* in this study. These results indicate that the mechanisms of Cd^2+^ transport, involving membrane transporter-related proteins, differed among pak choi cultivars.

### Cd transport regulatory networks in pak choi roots

The translocation of Cd in plants is mediated by transporters. Cd generally enters root cells through the transporters of Zn^2+^, Mn^2+^, Cu^2+^ and Fe^2+^ [7, 35]. Several genes encoding Cd-related transporters have been identified from *Arabidopsis* [12, 36, 37], rice [38], wheat [39] and tobaccos [40]. For example, Cd^2+^ can enter root cells through ZIP transporters, such as *ZIP2*, *ZIP3* and *IRT1* [28, 29]. These transporters have been implicated in mediated Cd influx into root cell across the plasma membrane of root epidermis cells [41, 42]. *IRT1* has also been described as an Fe transporter protein that is highly expressed in the cortex cells of *Arabidopsis* roots [42]. Plants with overexpressed *IRT1* accumulated higher levels of Cd and Zn than wild-type plants in *Arabidopsis* [43], suggesting that *IRT1* also mediates Cd^2+^ influx into root cells through the plasma membrane of the root epidermis and cortex cells. We found that *ZIP2*, *ZIP3* and *IRT1* are expressed at higher levels in Baiyewuyueman than in Kuishan’aijiaoheiye (Fig. 6; Table 3), implying that Baiyewuyueman has higher Cd concentration in roots than Kuishan’aijiaoheiye; however, no significant difference in root Cd concentration was observed between Baiyewuyueman and Kuishan’aijiaoheiye [25].

The heavy metal P_1B_-ATPases, such as *HMA2* and *HMA4* have been identified as efflux transporter genes responsible for the transport of Cd^2+^ from pericycle parenchyma cells to xylem [14, 15, 44]. These transportors have been localized on the plasma membrane and mainly expressed in the root pericycle cells [39, 45, 46]. In this study, the expression of *HMA2* and *HMA4* was up-regulated in Baiyewuyueman and down-regulated in Kuishan’aijiaoheiye in Cd treatments compared with the controls (Fig. 6 **;** Table 3). Similarly, RT-qPCR analysis revealed that Baiyewuyueman showed higher expression of *HMA2* and *HMA4* than Kuishan’aijiaoheiye under Cd treatment (Fig. 6). This observation is consistent with previous reports that the expression of *HMA4* in Cd-hyperaccumulating species was higher in roots and shoots compared with non-accumulators [47, 48].

Cd is often sequestered in the plant vacuole as Cd-chelate complexes [27, 49, 50]. Several tonoplast-localized transporters, such as *CAX4* [51], *HMA3* [37], *MTP3* [52, 53] and *MRP7* [31], are involved in Cd^2+^ transport into the vacuole. In *Arabidopsis*, *MTP3* has been confirmed to be involved in a Zn translocation, specifically expressed in epidermal and cortex cells of the root [53], and *HMA3* has been identified as involved in Cd translocation, with high expression levels in the guard cells, vascular tissues and root apex [37]. Moreover, the tonoplast-localized *AtHMA3* [54], *AtMRP7* [31], and *AtCAX4* [40] have been confirmed to enhance Cd tolerance of plants. RT-qPCR analysis indicates that the four unigenes encoding *CAX4*, *HMA3*, *MTP3* and *MRP7* have higher expression levels in Baiyewuyueman than in Kuishan’aijiaoheiye (Fig. 6a-j), implying the former had higher Cd tolerance than the latter. This result is consistent with our unpublished results that Baiyewuyueman has tolerance to high levels of Cd and rapid growth rate at the seedling stage under Cd treatment. In addition, *COPT5* has been identified as a copper export protein located in the tonoplast [55]. In *Arabidopsis*, Klaumann et al. [33] showed that *COPT5* T-DNA insertion results in decreased copper accumulation in siliques and seeds and increased copper accumulation in the vacuole of root cells. *COPT5* has also been confirmed to mediate Cd^2+^ efflux across the tonoplast of root cells into the cytosolic [36]. Our results show that the expression of *COPT5* is higher in Baiyewuyueman than in Kuishan’aijiaoheiye in KCd_10_ vs. BCd_10_, and down-regulated in KCd_10_ vs. KCd_0_ (Fig. 6 **;** Table 3). This finding is consistent with our previous study, implying that shoot Cd concentration was significantly higher in Baiyewuyueman than in Kuishan’aijiaoheiye [25]. Moreover, Cd induced down-regulation for *COPT5* and up-regulation for *MTP3* and *HMA3* in Kuishan’aijiaoheiye, suggesting that these genes might be responsible for enhancing Cd sequestration in the vacuole of root cells in Kuishan’aijiaoheiye. Additionally, many studies have confirmed that the protein systems excluding Cd from the cell cytosol localize to the plasma membrane and tonoplast are mediated by H^+^-coupled Cd antiport as well as by ATP-energized Cd pumps (e.g. H^+^-ATPase, V-ATPase and P-ATPase) [7, 28].

**Fig. 7:**
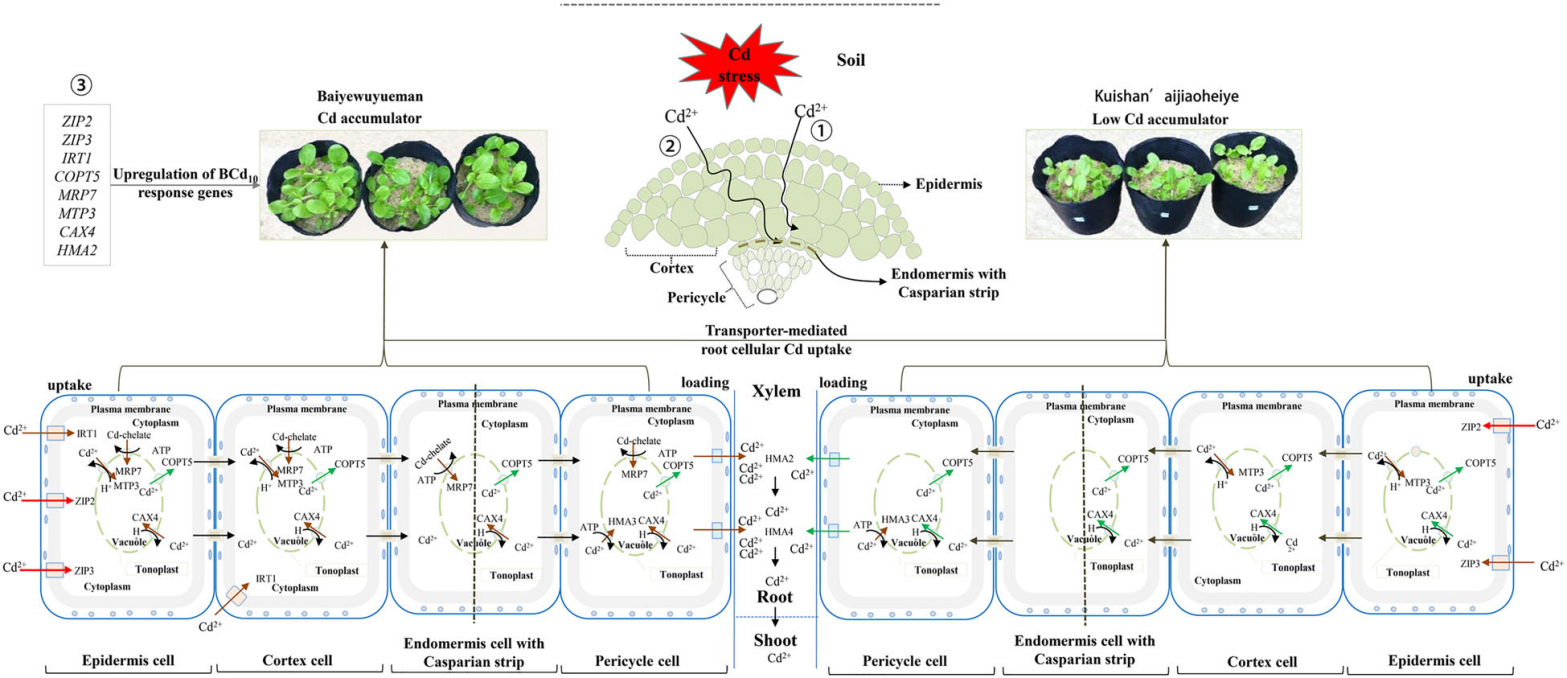
The putative model of regulatory networks associated with transport genes in the regulation of Cd translocation and compartmentation in pak choi roots. The apoplastic (1) and symplastic (2) pathways of Cd transport in pak choi roots; (3) The genes were identified as up-regulated in Baiyewuyueman compared with Kuishan’aijiaoheiye under Cd treatment by RNA-seq and RT-qPCR analysis; *red arrows* indicate the genes significantly up-regulated, *brown arrows* indicate the genes up-regulated, and *green arrows* indicate the genes down-regulated in Cd treatments compared with the controls by RNA-seq analysis. The localization of these genes was based on the data of Arabidopsis and rice (see Table 3)

Taken together, we proposed a schematic model to depict a regulatory network of root-to-shoot Cd translocation in pak choi (Fig. 7). The low transport rates of Cd into root epidermis cells from soil, and low loading rate of Cd into the xylem sap in the roots, are responsible for the low Cd translocation to shoots in Kuishan’aijiaoheiye. The findings could provide insights into the regulation of Cd translocation in pak choi, and facilitate further manipulation of low Cd accumulation genes in vegetable crops.

## Conclusions

The current study identified 6827 unigenes as DEGs in pairwise comparisons. Among them, 690 (BCd_10_ vs. BCd_0_), 2733 (KCd_10_ vs. KCd_0_), 2919 (KCd_0_ vs. BCd_0_) and 3455 (KCd_10_ vs. BCd_10_) play significant roles in response to Cd stress. Furthermore, 135 DEGs encoding ion transport related proteins (i.e., ZIPs, P_1B_-type ATPase and MTPs) were identified. Five plasma membrane-localized transport genes (*ZIP2*, *ZIP3*, *IRT1*, *HMA2* and *HMA4*) and five tonoplast-localized transport genes (*CAX4*, *HMA3*, *MRP7*, *MTP3* and *COPT5*), showing higher expression levels in Baiyewuyueman than in Kuishan’aijiaoheiye, might be responsible for cultivar differences in root-to-shoot Cd translocation in pak choi.

## Methods

### Plant material collection

Pot trials were conducted in a growth chamber with 16 h light at 25 °C and 8 h darkness at 16 °C. Based on the previous work [25], two pak choi cultivars, Baiyewuyueman (B, high-Cd cultivar) and Kuishan’aijiaoheiye (K, low-Cd cultivar), were used for the pot experiment. In order to avoid damaging the roots at harvest time, sand was used as the culture substrate. The washed sand was spiked with 0 (Cd_0_) and 10 mg/kg Cd (Cd_10_) as CdCl_2_•2.5H_2_O. The sand (1.2 kg) was filled in each pot (13 cm × 12 cm) and kept moist for 2 weeks prior to the experiment. Seeds were sown directly into pots. The treatments were arranged in a completely randomized design with six replicates (pots).

One week after sowing, the seedlings were thinned to six plants per pot. The plants were fertilized daily with 50 ml of nutrient solution as previously described [22]. Root samples for RNA-seq and RT-qPCR analysis were collected separately at 4 weeks after seedling emergence. Multiple independent biological replicates, each containing a pool of six different plants, were sampled for RNA-Seq (two biological replicates) and RT-qPCR validation (three biological replicates). All samples were immediately frozen in liquid nitrogen and stored at −80 °C.

### cDNA library construction and Illumina sequencing

Total RNA of root samples was isolated using Trizol® Reagent (Invitrogen) and purified using the RNeasy Plant Mini kit (Qiagen) according to the manufacturer’s instructions. A total of 3 μg RNA per sample was used for library preparation and sequencing. Eight cDNA libraries named B1_Cd_0_, B2_Cd_0_, B1_Cd_10_, B2_Cd_10_, K1_Cd_0_, K2_Cd_0_, K1_Cd_10_ and K2_Cd_10_ were constructed and sequenced as previously described [56]. The library construction and Illumina sequencing were conducted using the Illumina Hiseq™ 2500 platform following the manufacturer’s recommendations at Novogene Bioinformatics Institute (Beijing, China).

### Bioinformatic analysis

#### Data filtering

The raw reads in fastq format were initially processed with in-house Perl scripts. After filtering the adapter sequences, reads containing ploy-N and low-quality bases, clean reads were obtained. These clean reads were then used for further data analysis.

#### Transcriptome assembly

The left files (read 1 files) from all libraries/samples were pooled into one large left.fq file, and right files (read 2 files) into one large right.fq file. Transcriptome assembly was performed based on the left.fq and right.fq using Trinity [57] with min_kmer_cov set to 2 by default and all other parameters set as default.

#### Gene functional annotation and classification

The assembled unigenes were used in BLAST searches against public databases including NCBI non-redundant protein (Nr) and non-redundant nucleotide sequences (Nt), Protein family (Pfam), euKaryotic Ortholog Groups (KOG), Swiss-Prot, Kyoto Encyclopedia of Genes and Genomes (KEGG) pathway and Gene Ontology (GO) with an *E*-value cutoff of 10^−5^.

### Identification and functional enrichment analysis of DEGs

The clean reads were mapped back onto the assembled transcriptome reference sequences by RSEM [58] (no mismatch) for each sample. Next, the read count for each gene was obtained from the mapping results. Further, the expression level of each gene was normalized to FPKM (fragments per Kilobase per million fragments mapped) based on the number of readcount.

The DEGs were screened as previously described [56, 59]. Prior to differential gene expression analysis, the read counts for each sequenced library were adjusted by edgeR program package through one scaling normalized factor. Next, the average of readcount of the gene from two replicate libraries was calculated as the readcount value of the gene to analyze the differences among four groups: BCd_0_ (B1_Cd_0_ and B2_Cd_0_), BCd_10 (_B1_Cd_10_ and B2_Cd_10_), KCd_0_ (K1_Cd_0_ and K2_Cd_0_) and KCd_10_ (K1_Cd_10_ and K2_Cd_10_). The DESeq R package (1.10.1) was used to identify DEGs between two groups according to the method described by Anders & Huber [60]. DESeq provides statistical routines for determining differential expression in digital gene expression data using a model based on the negative binomial distribution. The resulting *P*-values were adjusted using the Benjamini and Hochberg’s approach for controlling the false discovery rate. A unigene found by DESeq with an adjusted *P*-value <0.05 was considered significantly differentially expressed.

Functional enrichment analysis was performed by the GOseq R packages based Wallenius non-central hyper-geometric distribution (corrected *P*-value <0.05) [61] and KOBAS (2.0) software (corrected *P*-value <0.05) [62]. Cluster analysis of gene expression patterns was performed with cluster 3.0 and Java Treeview software.

### RT-qPCR analysis

Total RNA was isolated as described above. First strand cDNA fragments were synthesized using the PrimeScript® RT reagent kit (Takara, Dalian, China). RT-qPCR was performed on an ABI 7300 (Applied Biosystems, Foster City, CA, USA) using a SYBR Premix EX Taq kit (Takara) in a 20 μl reaction mixtures, containing 2 μl of diluted cDNA, 0.2 μM forward and reverse primer, and 10 μl 2 × SYBR Green PCR Master Mix. The PCR reaction protocol was 95 °C for 30 s, 40 cycles of 95 °C for 5 s and 60 °C for 30 s. The fluorescence was measured via a 65–95 °C melting curve. All reactions were performed in triplicate with each replicate measured three times; the relative expression level of the selected unigenes using the *Actin* gene as the internal control gene was calculated using ratio = 2^−ΔΔ*C*τ^. The specific primers for RT-qPCR were designed using Beacon Designer 7.0 software (Premier Biosoft International, USA) and are listed in Additional file 6: Table S6.

## Additional files

Additional file 1: Table S1. KEGG pathways of the assembled unigenes in pak choi. (XLSX 239 kb)

Additional file 2: Table S2. Complete lists of differentially expressed genes in four groups based on pairwise comparisons. (XLSX 6819 kb)

Additional file 3: Table S3. The enriched GO terms of up and down-regulated DEGs in four comparisons. (XLSX 153 kb)

Additional file 4: Table S4. The significantly enriched pathways of up and down-regulated DEGs in four groups based on pairwise comparison. (XLSX 16 kb)

Additional file 5: Table S5. The DEGs involved in transport activities in two pak choi cultivars under Cd stress. (XLSX 84 kb)

Additional file 6: Table S6. The specific primer sequences of ten DEGs validated by RT-qPCR analysis. (XLSX 11 kb)

## Abbreviations

CAX: Cation exchanger
Cd: Cadmium
CML: Calcium-binding proteins
CNGC: Cyclic nucleotide gated channel
COPT: Copper transporter
DEG: Differentially expressed gene
GO: Gene ontology
HMA: Cadmium/zinc/copper-transporting ATPase (heavy metal-associated domain)
KEGG: Kyoto encyclopedia of genes and genomes
MRP: Multidrug resistance protein
MTP: Metal tolerance protein
Nramp: Natural resistance-associated macrophage protein
NRT: Nitrate transporter
OPT: Oligopeptide transporter
PDR: Pleiotropic drug resistance-type ABC transporter protein
RT-qPCR: Reverse-transcription quantitative PCR
ZIF: Zinc induced facilitator
ZIP: Zinc-regulated transporter/Iron-regulated transporter-like Protein
